# Longitudinal Observation of Outcomes and Patient Access to Integrated Care Following Point-of-Care Glycemic Screening in Community Health Center Dental Safety Net Clinics

**DOI:** 10.3389/froh.2021.670355

**Published:** 2021-05-26

**Authors:** Ingrid Glurich, Richard Berg, Aloksagar Panny, Neel Shimpi, Annie Steinmetz, Greg Nycz, Amit Acharya

**Affiliations:** ^1^Center for Oral and Systemic Health, Marshfield Clinic Research Institute, Marshfield, WI, United States; ^2^Office of Research Computing and Analytics, Marshfield Clinic Research Institute, Marshfield, WI, United States; ^3^Family Health Center of Marshfield, Inc., Marshfield Clinic Health System, Marshfield, WI, United States; ^4^Advocate Aurora Research Institute, LLC, Advocate Aurora Health, Inc., Downers Grove, IL, United States

**Keywords:** diabetes mellitus, prediabetic state, point-of-care testing, general practice, dental, glycated hemoglobin A, risk assessment, delivery of healthcare

## Abstract

**Introduction:** Rates of diabetes/prediabetes continue to increase, with disparity populations disproportionately affected. Previous field trials promoted point-of-care (POC) glycemic screening in dental settings as an additional primary care setting to identify potentially at-risk individuals requiring integrated care intervention. The present study observed outcomes of POC hemoglobin A1c (HbA1c) screening at community health center (CHC) dental clinics (DC) and compliance with longitudinal integrated care management among at-risk patients attending dental appointments.

**Materials and Methods:** POC HbA1c screening utilizing Food and Drug Administration (FDA)-approved instrumentation in DC settings and periodontal evaluation of at-risk dental patients with no prior diagnosis of diabetes/prediabetes and no glycemic testing in the preceding 6 months were undertaken. Screening of patients attending dental appointments from October 24, 2017, through September 24, 2018, was implemented at four Wisconsin CHC-DCs serving populations with a high representation of disparity. Subjects meeting at-risk profiles underwent POC HbA1c screening. Individuals with measures in the diabetic/prediabetic ranges were advised to seek further medical evaluation and were re-contacted after 3 months to document compliance. Longitudinal capture of glycemic measures in electronic health records for up to 2 years was undertaken for a subset (*n* = 44) of subjects with available clinical, medical, and dental data. Longitudinal glycemic status and frequency of medical and dental access for follow-up care were monitored.

**Results:** Risk assessment identified 224/915 (24.5%) patients who met inclusion criteria following two levels of risk screening, with 127/224 (57%) qualifying for POC HbA1c screening. Among those tested, 62/127 (49%) exhibited hyperglycemic measures: 55 in the prediabetic range and seven in the diabetic range. Moderate-to-severe periodontitis was more prevalent in patients with prediabetes/diabetes than in individuals with measures in the normal range. Participant follow-up compliance at 3 months was 90%. Longitudinal follow-up documented high rates of consistent access (100 and 89%, respectively), to the integrated medical/DC environment over 24 months for individuals with hyperglycemic screening measures.

**Conclusion:** POC glycemic screening revealed elevated HbA1c measures in nearly half of at-risk CHC-DC patients. Strong compliance with integrated medical/dental management over a 24-month interval was observed, documenting good patient receptivity to POC screening in the dental setting and compliance with integrated care follow-up by at-risk patients.

## Background

### Overview of Problem

The Centers for Disease Control and Prevention (CDC) projected that over 10.5% of individuals in the USA have diabetes mellitus (DM), with 21% undiagnosed [1]. Moreover, ~34.5% of the US adult population has prediabetes, with >80% unaware of their glycemic status (CDC, 2020) [2]. Between 2015 and 2030, diabetes prevalence in the USA is projected to increase by 54%, annual diabetes-associated mortality by 38%, and annual overall cost associated with diabetes to exceed $620 billion [3]. These data project that diabetes remains on track for continued escalation of its epidemic status.

Similarly, recently updated projections of periodontal disease (PD) reported increasing prevalence, currently estimated in excess of 40%, with higher rates projected among the elderly and in association with race and ethnicity, projected by population-based screening [4]. Because recent systematic review and meta-analysis of the evidence base surrounding bidirectional associations between PD and diabetes continues to support potential interactions between these conditions [5], there is an increased need to expand and promote integration of inter-disciplinary efforts across primary dental and medical settings to identify and manage high-risk individuals.

Whereas, current US Preventive Services Task Force (USPSTF) guidelines recommends screening for type 2 DM (T2DM) for individuals with hypertension, aged 40–70 years who meet obesity status definitions [6], glycemic screening in the dental setting has remained controversial (reviewed by Glurich et al. [7]). Biological screening in the dental setting was not recommended at the time USPSTF guidelines were issued because diabetes is not managed in the dental domain and an adequate evidence base to support screening was lacking. However, alignment of recent key developments supports timeliness of re-evaluation of dental clinic (DC) settings as primary care settings where at-risk patients can be identified. These key developments include (a) epidemiological evidence of the burgeoning epidemic status of T2DM and PD cited above; (b) evidence demonstrating substantive prevalence of undiagnosed T2DM/prediabetes in the DC setting [8, 9]; (c) publication of an expert consensus report and clinical guidelines recommending integrated T2DM and PD management issued by 2018 Joint Workshop International Diabetes Federation and European Federation of Periodontology following systematic examination of the evidence [10]; (d) findings of systematic review of meta meta-analyses surrounding bidirectional relationships between T2DM and PD, which continue to support value in integrated interventional approaches to prevention and treatment [11]; and (e) updated guidelines and issuance of Current Dental Terminology (CDT) codes (2019) to support point-of-care (POC) glycemic assessment in dental settings to inform patient management [12].

### Study Rationale

Implementation of POC hemoglobin A1c (HbA1c) screening across four community health center DCs (CHC-DC) in Wisconsin described herein was supported by a systematic review undertaken to examine outcomes of clinical and field trials published since 2007 exploring POC screening of patients attending dental visits [8]. Eligibility criteria for subjects enrolled in these field trials included the following: (1) no pre-existing diagnosis of T2DM/prediabetes, (2) no biological glycemic measure in a defined period, and (3) documentation that patients had known risk factors for diabetes [8]. These studies sought to estimate prevalence of undiagnosed T2DM/prediabetes in their dental patient population. Substantial rates of putative T2DM (1–14%) and prediabetes (19–90%) were detected across a range of dental practices with highest rates observed in dental practices serving a higher proportion of patients meeting disparity population definitions [13, 14]. However, studies that reported on re-evaluation of glycemic measures in the medical setting on patients testing into hyperglycemic ranges mainly did so only within 24–48 h following the POC screening test and failed to establish the true rate of diabetes diagnosis based on the prevailing clinical practice guidelines effective in the temporal window of these studies. These guidelines stated requirements for confirmatory glycemic measures in the diabetic range within 6 months. Longitudinal follow-up of further glycemic evaluation or concordance between the screening measures and further biological glycemic assessment across time was also not an objective of the field trials. Furthermore, instrumentation to conduct biological glycemic screening varied across studies and included glucometers not approved by the Food and Drug Administration (FDA) for global screening in 7/10 studies systematically reviewed [8]. Finally, the glycemic measure used to screen glycemic levels at POC also varied across studies with 7/10 employing HbA1c [8]. Findings of the systematic review underlined a need for appropriately designed protocols to support further assessment of the relative clinical value in conducting POC screening in the DC setting. Emphasis was placed on targeting of undiagnosed patients with risk factors for DM and no glycemic measures within a defined temporal window in order to evaluate the value of designating the DC setting as an additional interdisciplinary primary care setting.

Analysis of longitudinal patient engagement in integrated care delivery following POC screening was also of interest to CHC-DC operationalizing safety net operations. In lieu of population-based screening, targeted screening was posited to identify potentially undiagnosed individuals who require further medical assessment and appropriate follow-up in both the medical and dental settings. Notably, regression modeling of candidate variables contributing to diabetic risk by authors of previous field trials screening for undiagnosed hyperglycemia in DC settings identified PD prevalence and missing teeth as novel independent risk factors [13, 14]. Detection of T2DM/prediabetes risk, ideally at early stages, was targeted positing that intervention during early development could slow or prevent progression in activated patients and potentially reduce risk for onset of diabetic complications and chronicity of PD.

The focus of the current study was to implement POC HbA1c testing to detect rates of T2DM and prediabetes across patients of four CHC-DC in Wisconsin with targeted screening only of the subset of patients with risk factors for hyperglycemia and observe patient behavior relative to seeking medical–dental access if glycemic screening measures were elevated. The study design included questionnaire-based screening, in combination with Clinical Laboratory Improvement Act (CLIA)-waived HbA1c testing utilizing Federal FDA-approved instrumentation in the DC settings targeting only dental patients with high-risk profiles. Observational longitudinal follow-up was further planned to monitor patient compliance with triage and follow-up testing by medical providers 3 months post-screening. Finally, a subset of patients across three of four centers where data were accessible in the electronic health records (EHRs) was monitored for glycemic follow-up and evidence of periodontal evaluation within a minimum time frame of 1 year and up to 2 years post-POC screening in order to more accurately observe concordance of screening outcome, true biological status, and access to available integrated medical/dental care delivery models in CHC settings.

## Design and Methods

### Study Design and Objectives

**Figure 1:**
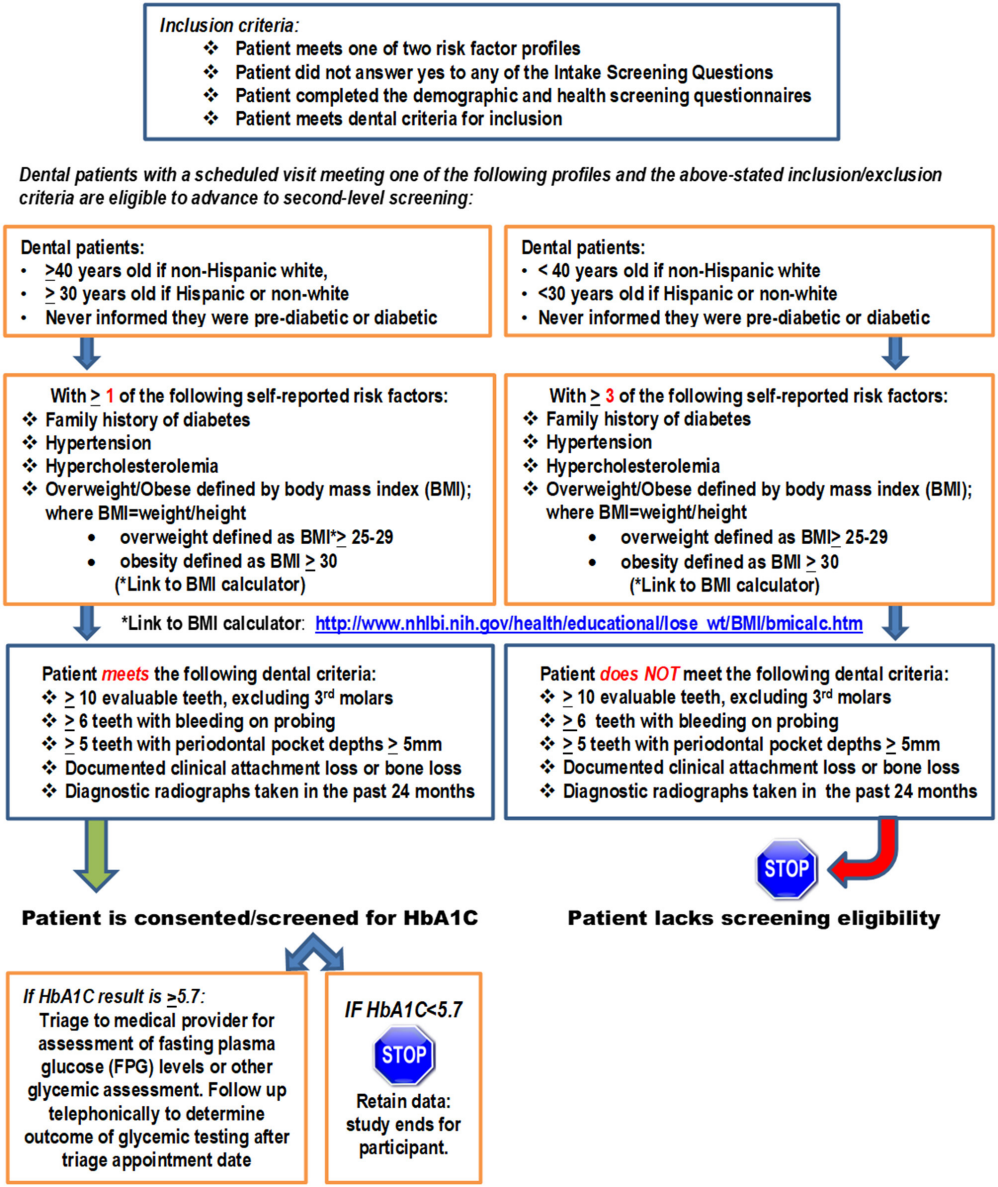
Study flow diagram for study-eligible dental patients.

This observational community case study evaluated clinical utility of identifying the subset of eligible dental patients potentially at risk for T2DM/prediabetes in the context of scheduled dental visits at participating CHC-DC sites where a POC HbA1c screening protocol was implemented. Specifically, the study focused on the subset of individuals attending dental care appointments with no existing diagnosis or history of DM/prediabetes and no glycemic screening within the past 6 months to document glycemic status but who exhibit risk factors for diabetes and met inclusion criteria as outlined in the study flow diagram in Figure 1.

The study objectives included observational characterization of (1) undiagnosed dysglycemia prevalence detected; (2) tracking of compliance with triage to medical evaluation and follow-up; and (3) longitudinal tracking of individuals to observe access to medical and dental care for individuals found to be at high risk for T2DM/prediabetes following POC screening in the dental setting.

### Population and Setting

This community case study was undertaken across three CHC-DC serving largely rural populations in Wisconsin including 2 of 10 CHC-DC operated by Family Health Center of Marshfield, Inc. (FHC-M) and Marshfield Clinic Health System (MCHS) in the following: (1) Marshfield, Wisconsin (WI); (2) Medford, WI; and (3) Bridge Community Dental Center serving the regional population of Wausau, WI. The fourth DC that enrolled patients was St. Elizabeth Ann Seton Dental Clinic, a walk-in clinic serving an urban population in Milwaukee, WI. Study enrollment was undertaken over 11 months from October 24, 2017, through September 24, 2018. Longitudinal follow-up was carried out for a minimum additional 12 to up to 24 months through October 1, 2019, on subjects with available data in order to observe patient longitudinal access for follow-up glycemic measures in the medical setting and periodontal assessments in the dental setting. All of the participating sites represent DCs designated as dental safety nets established largely in rural settings to serve disparity populations who otherwise have limited access to dental care [15]. Over 85% of patients seeking care at FHC-M dental centers alone are on Medicaid [16] and other CHCs similarly provide dental care to a high volume of the Medicaid population and to those with no dental insurance coverage largely due to poverty status. The majority of patients seen at St. Elizabeth Ann Seton Dental Clinic in Milwaukee, as a “walk-in” clinic, have no dental home. Their operations largely target provision of dental care to individuals experiencing acute dental conditions. While periodontal assessment and longitudinal tracking data on these patients were not available, patient enrollment at this fourth site was included mainly to explore rates of hyperglycemia in their patient population and gauge receptivity of clientele of this clinic to HbA1c screening in the dental setting.

### Overview of Participant Screening Procedures

The study and all study forms were reviewed and approved by the Institutional Review Board of the MCHS. Participating DCs applied for and were issued CLIA waivers to support conduct of HbA1c screening in the dental setting using Siemens DCA Vantage HbA1c Analyzer (Siemens Healthineers, USA). This analyzer uses an immuno-assay to determine HbA1c measurement, has FDA approval for CLIA-waived POC HbA1c screening in the clinical setting, and reproduces results of the validated laboratory reference method HA 8160 cationic exchange high-performance liquid chromatography with high fidelity and accuracy [17]. The instrument was supplied to the study team through the 17VNPL DCA Vantage Analyzer Placement Program [18]. All testing supplies, reagents, and normal/abnormal controls were purchased from the manufacturer.

Sample size estimates were based on targeting of 1,000 patients for initial screening to identify patients with undiagnosed diabetes/prediabetes based on rates reported in earlier field trials examining POC glycemic testing in the dental setting [8] and estimated patient census across the four sites. Enrollment of ~200 undiagnosed cases was conservatively projected. Enrollment was terminated at 11 months following screening of 915 patients and enrollment of the study-eligible cohort (*n* = 224). Screening was accomplished in two steps. The *Intake Screening Questionnaire* consisting of nine questions was completed by all patients presenting at the participating dental centers to determine eligibility for POC HbA1c screening in the dental setting (Appendix 1). Those who answered “no” to all questions met eligibility criteria and gave written informed consent for enrollment in the study. Enrolled subjects next completed the demographic and comorbidity profile questionnaire (Appendix 2), which consisted of 12 questions, and the American Diabetes Association Diabetes risk test (https://www.diabetes.org/risk-test), which generated a risk score. The final screening step involved capillary collection of blood following a finger stick and analysis of the HbA1c measure by the Siemens DCA Vantage HbA1c Analyzer. Enrolled subjects with measures < 5.7% exited the study, while those with measures ≥ 5.7% were continued for longitudinal observational follow-up for at least 12 months. These subjects first received telephonic follow-up 3 months following HbA1c screening to determine whether they had complied with recommended triage to medical providers for further monitoring of their glycemic status. Follow-up included longitudinal tracking of glycemic measure outcomes or identification of a new prescription for medications associated with glycemic regulation in the medical setting and observation of periodontal assessments and/or other dental procedures in the dental setting. Patients also either underwent periodontal assessment at time of enrollment or had clinical assessments abstracted from the EHRs if they had been evaluated within 3 months of study enrollment. Assessment criteria included documentation of bone loss, attachment loss, and moderate-to-severe PD based on updated definitions of PD classification defined by the American Academy of Periodontology (AAP) Task Force [19]. Figure 1 summarizes parameters applied to ensure stringency regarding documentation of PD. Patients were required to have a minimum of 10 evaluable teeth excluding third molars. Further requirements included documentation of the following: ≥6 with bleeding on probing, ≥5 teeth with periodontal pocket depth (PPD) ≥ 5 mm, and evidence of clinical attachment loss ≥ 3 mm or >16% (≥3-mm bone loss) based on diagnostic radiographs captured within the past 24 months as defined by AAP classification definitions [18]. Data on number of missing teeth were also collected.

### Analytical Approach

Data were summarized to characterize participant characteristics and study outcomes relative to glycemic measures. HbA1c values defined by American Diabetes Association were used to classify normal range (<5.7%), prediabetic range (≥5.7–6.4%), and diabetic range (>6.4%) [20]. Outcomes of study subjects with measures ≥ 5.7% and rate of access to medical and dental care and glycemic measures captured in the EHR were also tracked over time to determine integrated care access. Due to small numbers of subjects with measures in the diabetic range, these patients were pooled with those in the prediabetic range for statistical comparisons Fisher's exact test was used for comparisons of categorical characteristics (e.g., gender), and the Wilcoxon rank sum test was used for comparisons of numerical characteristics (e.g., age).

## Results

### Population Characteristics

Across the four CHC-DC sites, a total of 915 patients were initially approached to identify 224 (24%) with no existing diagnosis of T2DM/prediabetes or glycemic evaluation in the past 6 months. Following exclusion of four individuals, 127/220 (58%) met criteria for potential risk for undiagnosed T2DM/prediabetes and underwent further screening and POC HbA1c testing. Characteristics of the screened cohort are summarized in Table 1. Screening for risk factors selected a cohort characterized by older age and higher frequency of Hispanic ethnicity.

**Table 1:** Descriptive characteristics of screened cohort.

	**Study eligible?**	
	**No (*n* = 93)**	**Yes (*n* = 127)**
Mean age (years)	32.3 ± 9.5	51.1 ± 13.3
Male	30.1%	35.4%
White race	76.3%	68.5%
Hispanic	6.5%	19.7%
Hypertension	6.5%	35.4%
Hypercholesterolemia	2.2%	36.2%
Mean BMI	28.5 ± 8	30.9 ± 7.9
History of smoking	59.1%	49.6%

*BMI, body mass index*.

Among study-eligible subjects (*n* = 127), 100% underwent POC HbA1c screening. Results of HbA1c shown in Table 2 found that 62/127 (49%) of the subset of potentially at-risk patients had POC screening HbA1c values ≥ 5.7%, with 55/62 (89%) and 7/62 (11%), exhibiting measures in the prediabetic and diabetic ranges, respectively. Subjects with HbA1c measures above normal ranges were somewhat older and showed some differences in established risk factors, but our numbers in the diabetic range were too small (*n* = 7, with HbA1c > 6.4%) for definitive comparisons.

**Table 2:** Outcomes of POC HbA1c screening summarized by participant characteristics.

	**Normal^#^**	**Pre-DM^##^**	**DM^###^**	
	**(*n* = 65)**	**(*n* = 55)**	**(*n* = 7)**	***P*-value** ^a^
Mean age (years)	48.9 ± 13.3	53.5 ± 13.5	51.9 ± 9.5	0.035
Male	30.8%	34.5%	85.7%	0.273
White race	70.8%	65.5%	71.4%	0.703
Hispanic	18.5%	20.0%	28.6%	0.824
Hypertension	36.9%	30.9%	57.1%	0.853
Hypercholesterolemia	35.4%	34.5%	57.1%	0.856
Mean BMI	29.9 ± 8.5	31.8 ± 7.3	32.8 ± 6.0	0.091
History of smoking	58.5%	40.0%	42.9%	0.051

a *Test result comparing normal to pre-DM pooled with DM. Percentage of participants meeting PD definitions is shown for each subset of patients classified by HbA1c screening outcomes reflecting glycemic status as defined by the American Diabetes Association: normal range*

# *(< 5.7%), prediabetic range*

## *(≥5.7–6.4%), and diabetic range*

### *(>6.4%) [19]. Data in this table were based on self-reported responses completed by eligible participants at time of enrollment in response to the questionnaires (see Appendices)*.

*POC, point-of-care; HbA1c, hemoglobin A1c; DM, diabetes mellitus*.

### Observations Across Dental Variables

Among enrolled subjects, periodontal assessments within 3 months or at time of enrollment were captured for 100/127 (79%) of at-risk subjects who underwent POC HbA1c screening in dental settings. Table 3 shows outcomes of the periodontal assessment stratified by glycemic status indicated by outcome of POC HbA1c screening measures, including percent of subjects with bone loss, attachment loss, and moderate-to-severe PD based on updated definitions of PD classification defined by the AAP Task Force [19]. Although the differences were not statistically significant, subjects with elevated HbA1c measures showed higher levels of PD than those with normal measures across all three periodontal parameters assessed.

**Table 3:** Dental measures as available for cohort with POC HbA1c screening data.

	**Normal^#^**	**Pre-DM^##^**	**DM^###^**	
	**(*n* = 50)**	**(*n* = 45)**	**(*n* = 5)**	***P*-value^a^**
Attachment loss	76.6%	82.5%	100%	0.434
Bone loss	70.2%	85.7%	80.0%	0.136
Moderate/severe PD	31.1%	43.6%	80.0%	0.132
Mean number of missing teeth	5.4 ± 6.4	5.7 ± 6.0	3 ± 2.2	0.777
Mean bleeding on probing	5 ± 6.8	4.4 ± 5.3	11 ± 9.0	0.660

a *Results of statistical evaluations comparing normal with pre-DM pooled with DM. Definitions of criteria used to define moderate-to-severe PD for study participants: patients were required to have a minimum of 10 evaluable teeth excluding third molars. Furthermore, documentation of the following parameters was required: ≥6 teeth with bleeding on probing, ≥5 teeth with periodontal pocket depths (PPDs) of ≥5 mm, evidence of clinical attachment loss ≥ 3 mm, or ≥16% (≥3 mm) bone loss based on diagnostic radiographs captured within the past 24 months, as defined by AAP classification definitions [18]. Data on number of missing teeth were also captured. Percentage of participants meeting PD definitions is shown for each subset of patients classified by HbA1c screening outcomes reflecting glycemic status as defined by the American Diabetes Association: normal range*

# *(< 5.7%); prediabetic range*

## *(≥5.7–6.4%); and diabetic range*

### *(>6.4%) [19]*.

*POC, point of care; HbA1c, hemoglobin A1c; DM, diabetes mellitus; AAP, American Academy of Periodontology; PD, periodontal disease*.

### Longitudinal Follow-Up

**Figure 2:**
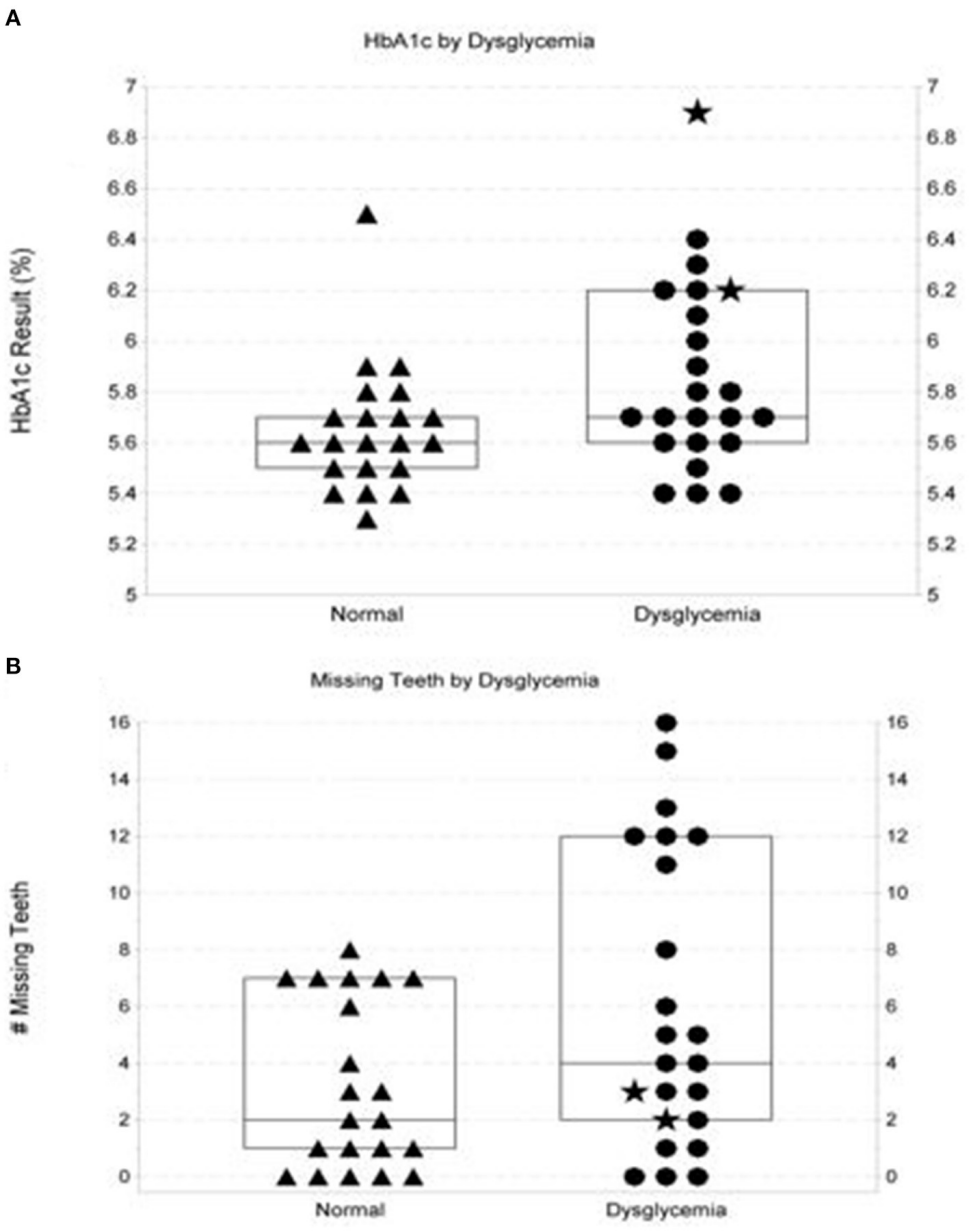
Characteristics of at-risk dental patients with normo-glycemic point-of-care screening outcomes vs. those with outcomes in the pre-diabetic/diabetic range. **(A)** shows distribution of follow-up glycemic measures performed by a commercial laboratory in the medical setting on at-risk patients with normoglycemic vs. elevated glycemic measures when screened at point-of-care. Box plot in **(A)** show the median and interquartile range. Two patients screening in the diabetic range are denoted by ⋆. The figure shows a trend (*p* = 0.054) for more follow-up measures in hyperglycemic ranges (defined as HbA1c measures ≥ 5.7% or fasting plasma glucose measures ≥ 100 mg/dL. **(B)** illustrates observations surrounding numbers of missing teeth documented in at-risk patients with initial elevated HbA1c screening measures (≥5.7%) vs. number of missing teeth in those with point-of-care HbA1c measures in the normoglycemic range (*p*-value = 0.094). Box plots in **(B)** show the median and interquartile range. Two patients screening in the diabetic range are denoted by ⋆.

At 3 months, 90% of subjects who had undergone biological screening with HbA1c measures ≥ 5.7% participated in telephonic follow-up. At follow-up, 79% reported having attended or scheduled appointments with medical providers. Longitudinal follow-up for ≥12 months (range: >12–24 months) by monitoring glycemic measures and prescription data for pharmaceuticals targeting glycemic control was possible for 44/127 (35%) of subjects enrolled at FHC-M dental centers or the Bridge Community site, who also accessed medical care through MCHS. As shown in Figure 2A, mean glycemic measures determined in the medical setting in patients with HbA1c measures in the normal range captured at POC in the dental setting were lower than mean of measures for those subjects whose screening measure captured at POC in the dental setting was ≥5.7% (5.6 vs. 6.2%, respectively). A trend toward higher prevalence of missing teeth was also noted among those with POC HbA1c measures ≥ 5.7% (Figure 2B).

The integrated medical–dental EHR (iEHR) was screened from time of enrollment to up to 24 months to capture any new laboratory data indicating glycemic screening. During the 24 months of follow-up, 153 glucose measures across the 42 patients were documented in the iEHR (mean = 3.6 measures per patient; range: 1-16 measures). Comparing results of POC HbA1c measures at time of enrollment and at time of first follow-up glycemic measure (HbA1c or fasting plasma glucose), elevated glycemic status at screening was corroborated in 32/44 (73%) of subjects. Notably, fasting and random glucose measures were more routinely performed to monitor at-risk patients and were available for 42/44 patients. For two patients, only pharmaceutical exposures to medications indicating glycemic management were available for follow-up. Observation of glycemic data for >12 months (up to 24 months) was possible for 29/42 (69%) of subjects being followed up for whom laboratory values were available. Among 4/127 participants (3.1%), a new diagnosis of T2DM was validated based on confirmation of glycemic measures during longitudinal follow-up, assignment of new diagnostic code, and/or newly prescribed medications for glycemic control. Among the 23/44 patients with screening measures in the prediabetic range for whom longitudinal follow-up was possible, prediabetic/diabetic status was validated in 18/23 (78%) of subjects during longitudinal follow-up. An additional six patients who had exhibited high-normal values for POC HbA1c screening measures were found to have measures in the prediabetic range during follow-up.

A trend toward improved glycemic status over time was noted in 20% of subjects in response to pharmacological management and/or lifestyle changes. Patient access for dental management was also trackable for 80% of 44 patients with available data in the EHR. Among these patients, 88% underwent at least one periodontal examination during the 2-year observational follow-up window.

## Discussion

### Findings Regarding Rate of Hyperglycemic Risk in the Community Health Center Dental Clinic Population

A growing evidence base continues to support that onset and progression of chronic systemic and oral diseases are driven by integrated pathophysiological processes and impact on health outcomes in a holistic manner. In this scenario, simultaneous exacerbation may occur with bidirectional contributions impacting both oral and systemic disease severity especially in the absence of effective integrated intervention. Increasingly, re-evaluation of our health-care delivery models has been advocated with emphasis on evolution of improved integrated medical–dental care delivery models supported by systematic examination to show evidence that such models are cost-effective and actually leverage improved patient outcomes [21]. In the absence of medical–dental integration across the entire spectrum of stakeholders, which may be further confounded by disparities in access experienced by some segments of the population, the potential for contribution to the epidemic escalation of diabetes and PD remains. The current study sought to examine whether one targeted intervention, namely, biological testing for glycemic status in the unconventional primary DC setting that provides health care to populations with overrepresentation of disparity populations, could activate patients to access care by providers practicing in an integrated care delivery environment. Among study-eligible, high-risk subjects with a low track record for glycemic monitoring attending dental visits at CHC-DC who underwent HbA1c screening at POC, the rate of hyperglycemia was 49%. Notably, as seen in Table 1, a higher percentage of study-eligible subjects reported comorbidities [hypertension, hypercholesterolemia, and body mass index (BMI) > 30] compared with the screened population whose risk profile did not meet eligibility requirements for POC screening, corroborating previously reported findings regarding high prevalence of multiple comorbidities among patients with diabetes [22]. Moreover, in the subpopulation with elevated glycemic screening measures where longitudinal follow-up was possible, screening results were corroborated for 78% of participants.

### Outcomes of Longitudinal Follow-Up

This case study examined longitudinal follow-up in the longest temporal window reported to date (up to 2 years) following implementation of POC glycemic screening in four CHC dental primary care settings to determine impact on patient care-seeking behavior in health-care environments offering integrated care delivery access in the context of dental safety net operations. Implementation of biological screening for hyperglycemia at POC using FDA-approved glucometers in the subpopulation of appointed dental patients meeting high-risk profiles detected a 24% rate of at-risk individuals based on patient survey responses alone. Among this subset, 58% qualified for POC HbA1c screening in the dental primary care setting. Notably, differences surrounding periodontal prevalence in this relatively small study between patients screening in the normoglycemic and hyperglycemic were not statistically significant. Nonetheless, a trend toward higher rates of more advanced PD was noted among subjects with POC HbA1c measures in the prediabetic and diabetic ranges as compared with patients with normoglycemic measures as evidenced by percent of bone loss, attachment loss, and PD severity level across the glycemic strata. Telephonic follow-up at 3 months to monitor subjects' planned compliance with recommended triage for follow-up with a medical provider was possible for 90% of all study participants. Among these participants, all but one patient indicated compliance or planned follow-up. Notably, among the subset of participants where investigators could access data in the iEHR, semi-annual or annual glycemic assessments for up to 2 years post the date of POC screening were documented for 100% of subjects. Moreover, attendance for annual periodontal assessments up to 24 months post-POC testing was also documented for 80% of the subset. Taken together, longitudinal observation documented a change in patient behavior relative to accessing integrated care for glycemic and periodontal assessment, and a high level of patient activation following POC glycemic screening in the CHC-DC setting was observed.

### Comparisons With Historical Field Trials Examining Point-of-Care Glycemic Testing in Community Health Center Dental Clinic Settings

In a field trial conducted by Genco et al. [23] that examined feasibility of POC glycemic screening across a range of dental settings, the authors similarly observed that compliance with triage for glycemic monitoring in the medical setting was highest in the CHCs compared with private dental practices (79 vs. 22% (*p* = 0.001). Furthermore, 85% compliance was noted in a CHC with an integrated care delivery model participating in their field trial [23]. Data from the current study corroborate initial findings reported by Genco et al. Notably, Greenberg et al. [24] also found higher rates of acceptance for triage to the medical setting by dental providers among patients attending DCs (86%) vs. private dental practices (76%). A systematic review examining the role of diabetic screening in the dental setting by various stakeholders similarly reported that five studies examining patient opinion surrounding acceptability of diabetic screening in the dental setting unanimously reported high rates of acceptability [25].

PD represents an early complication and harbinger of diabetes/prediabetes [9], emphasizing the need for cross-disciplinary integrated care delivery models. A 2015 study conducted in an outpatient clinic serving low- to mid-income population in Amsterdam treating patients with diabetes in a non-integrated setting conducted a trial targeting improved communication between medical and dental professionals. An alternative model explored in an additional study by these authors included provision of an oral health questionnaire completed by the dentist, and periodontal screening index (PSI) score was supplied to the physician during patient visits to inform patient management as an alternative approach to POC testing. Notably, among patients with moderate-to-high PSI scores, 65% had untreated PD. The study reported that moderate-to-high PSI was moderately more prevalent in 54% of the population with T2DM and in 57% of patients exhibiting obesity, but response rate for questionnaire completion was reported as 41% [26].

These data suggest that some populations may be more responsive to accessing integrated care delivery, although reasons for this are currently unclear and would require further investigation. Given that CHC-DC serve disparity populations with among the highest rates of PD and diabetes, data from the current study and other initial field trials suggest that the clientele of CHC-DC operating as safety nets are motivated to access integrated care delivery that offers affordable access to both medical and dental care for this population. Moreover, such populations are likely to derive the greatest level of benefit given the high prevalence of PD and diabetes documented among disparity populations. However, due to limited sample sizes of studies to date that have been able to observe integrated care access, studies in larger populations across more diverse populations are needed to further test this premise.

### Study Limitations

Some study limitations are noteworthy. Longitudinal glycemic and dental follow-up was possible for approximately one third of 44/127 (35%) of study participants who underwent HbA1c screening and was not possible for an additional 35% of patients seen mainly for treatment of dental emergencies at the walk-in St. Elizabeth Ann Seton Dental Clinic in Milwaukee Wisconsin, which does not provide routine dental care to these patients or track their dental history. However, the walk-in St. Elizabeth Ann Seton Dental Clinic patients were responsive to study participation, and 20/44 (45%) of them indicated intent to comply with recommendation for medical follow-up. For the remaining 30% of patients, access to longitudinal follow-up data was not available, although these patients may have sought care in other health-care systems where EHR access was not possible. Whereas, glycemic evaluation for fasting or random glucose measure in the medical setting was available for 95% of patients, follow-up HbA1c measures were only available for 62% of participants. Finally, due to constraints in the sample size in which longitudinal follow-up was possible, further data modeling was precluded.

## Data Availability Statement

The raw data supporting the conclusions of this article will be made available by the authors, without undue reservation.

## Ethics Statement

The studies involving human participants were reviewed and approved by Marshfield Clinic Health Systems Internal Review Board. The patients/participants provided their written informed consent to participate in this study.

## Author Contributions

IG contributed to study design, drafted the study protocol, data analysis, regulatory paperwork, implemented point-of-care glycemic testing and protocols, and drafted the manuscript. RB performed biostatistical analyses, informed statistical aspects of study design, and edited the manuscript. AP assisted with data management, data set preparation, data analysis, and reviewed manuscript. NS contributed to strategic design of all study objectives, final review, final editing of manuscript, and figure development. AS provided day-to-day oversight of study activities, tracked enrollment, quality checked data entry, developed reports, provided oversight of multi-site project management, fiscal oversight, and review the final manuscript. GN participated in study design relative to operational aspects occurring in the dental clinical setting, facilitated establishment and integration of point-of-care glycemic testing and research activities involving patient examinations into clinical operations, championed the study with dentists and hygienists, and participated in final editing of manuscript. AA initiated the study, obtained and oversaw funding for study support, provided study oversight, lead the study design and activities of the research team, prepared study reports, and participated in final editing of the manuscript. All authors contributed to the article and approved the submitted version.

## Conflict of Interest

The authors declare that the research was conducted in the absence of any commercial or financial relationships that could be construed as a potential conflict of interest.
