# Supplementary material for: Longitudinal Observation of Outcomes and Patient Access to Integrated Care Following Point-of-Care Glycemic Screening in Community Health Center Dental Safety Net Clinics
Source: Front Oral Health. 2021 May 26;2:670355. doi: 10.3389/froh.2021.670355 (PMC8757706; doi:10.3389/froh.2021.670355)
Supplement: Supplementary file 1 [file Table_1.DOCX]

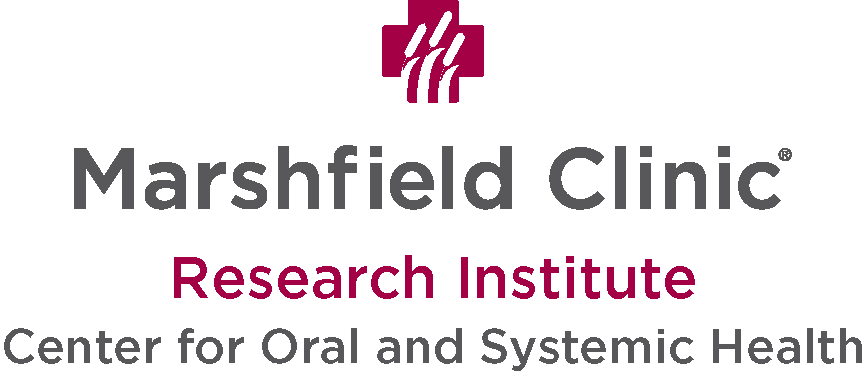


715-389-4460

1-800-782-8581

Fax 715-221-6402

**Study Title:** *A multi-site field trial to establish a screening and referral protocol for dental patients with undiagnosed diabetes/prediabetes*

**Appendix 1: Eligibility Intake Screening Questionnaire**

**Do any of the following relate to your health?** *(Check all that apply)*

|  | Yes | | No | |
| --- | --- | --- | --- | --- |
| Are you younger than 21 years of age? | |  | |  |
| Are you pregnant? | |  | |  |
| Have you taken antibiotics in the past 6 months? | |  | |  |
| Have you had your blood sugar level tested in the past 6 months? | |  | |  |
| Has a physician told you that you are diabetic? | |  | |  |
| Do you have less than 10 teeth in your mouth? | |  | |  |
| Do you have severe cardiovascular (heart) disease? | |  | |  |
| Do you have liver disease? | |  | |  |
| Do you have anemia or any blood disorders? | |  | |  |

If you are not sure if you have any conditions listed in the box, please select ‘no’.

*Thank you for completing this form.*
