# Supplementary material for: Longitudinal Observation of Outcomes and Patient Access to Integrated Care Following Point-of-Care Glycemic Screening in Community Health Center Dental Safety Net Clinics
Source: Front Oral Health. 2021 May 26;2:670355. doi: 10.3389/froh.2021.670355 (PMC8757706; doi:10.3389/froh.2021.670355)
Supplement: Supplementary file 2 [file Table_2.DOCX]

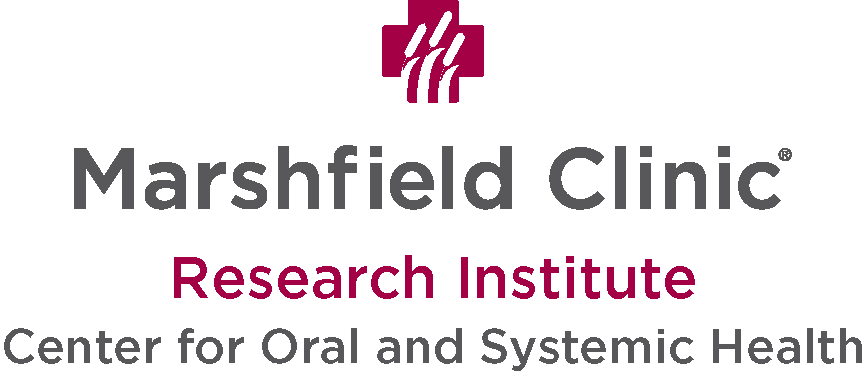


715-389-4460

1-800-782-8581

Fax 715-221-6402

Study #: ______________ *(BCW01, etc.)*

­­

**Study Title:** *A multi-site pilot effort to establish a screening and referral protocol for dental patients with undiagnosed dysglycemia*

**Demographic Data & Self-Reported Health Questionnaire**

|  | Yes | | No |
| --- | --- | --- | --- |
| 1) Do you usually have daily physical activity of at least 30 minutes at work, or during leisure time including normal daily activities? | |  |  |
| 2) Have you, or do you currently take medications for blood pressure control? | |  |  |
| 3) Do you have high cholesterol? | |  |  |
| 4) Do you take medications to control your cholesterol levels? | |  |  |
| 5) Have you ever been found to have high blood glucose during a health examination, pregnancy or illness? | |  |  |
| 6) Have you ever smoked cigarettes? | |  |  |
| 7) Are you a current smoker? | |  |  |
| 8) What is your gender? | | - Female | - Male |
| 9) If you are a female who had children, did any of your infants weigh 9 pounds or more at birth? | |  |  |
| 10) Race: | | - White - Black - Asian - Native American - Multi-racial |  |
| 11) Ethnicity: | | - Hispanic | - Non-Hispanic |
| 12) Please provide your birth year: ___________ | |  |  |

***Please turn paper over and complete Page 2 of questionnaire 🡪***

**Demographic Data & Self-Reported Health Questionnaire**

We are requesting the following information because exposure to medications and certain disease conditions may cause inaccurate measures of you glycemic levels. Please provide the following information:

Are you taking medications? If so, please list medication and the reason for taking it:

| **Name of Medication** | **Reason** |
| --- | --- |
|  |  |
|  |  |
|  |  |
|  |  |
|  |  |
|  |  |
|  |  |
|  |  |

Do you have:

Health Insurance coverage?

🞏Yes 🞏No

Medicare/Medicaid coverage or other subsidized insurance coverage?

🞏Yes 🞏No

Dental insurance coverage?

🞏Yes 🞏No
